# Supplementary material for: Clinical Outcomes in Patients Aged 80 Years or Older Receiving Non-Invasive Respiratory Support for Hypoxemic Acute Respiratory Failure Consequent to COVID-19
Source: J Clin Med. 2022 Mar 2;11(5):1372. doi: 10.3390/jcm11051372 (PMC8911338; doi:10.3390/jcm11051372)
Supplement: Supplementary file 1 [file jcm-11-01372-s001.zip › jcm-1612786-supplementary.pdf]

Supplementary materials

Treatment algorithm

A similar treatment algorithm, based on a stepwise utilization of High Flow Nasal Oxygen, Continuous Positive Airway Pressure (CPAP) or Non-Invasive Ventilation (NIV), and endotracheal intubation (ETI) was utilized by participating Intermediate respiratory care units in the effort to reverse hypoxemia in patients with COVID-19 [1]. The decision to proceed to CPAP or NIV was taken by the attending physician depending on local training habits.

[1] Pasin L, Sella N, Correale C, et al. Regional COVID-19 Network for Coordination of SARS-CoV-2 outbreak in Veneto, Italy. J Cardiothorac Vasc Anesth. 2020;34(9):2341-5.

Continuous positive airway pressure

Continuous positive airway pressure (CPAP) was delivered through helmets (Intersurgical, Mirandola (MO), Italy; Dimar, Medolla, MO, Italy) and face masks (Intersurgical, Mirandola, MO, Italy; Dimar, Medolla, MO, Italy; Fisher&Paykel, Auckland, New Zealand; ResMed, San Diego, CA, USA; Philips Respironics, Murrysville, PA, USA) via Boussignac systems or via flow-meters (typically 30-50 l/min depending on the interface chosen), using a scale that allowed clinicians to regulate oxygen and air flow separately to set inspiratory oxygen fraction (FiO_2). A bacterial and viral filter was applied to the expiratory port. CPAP was set between 10 and 12 cmH_2O according to the patient's needs, tolerance, and any side effects. CPAP pressure could be increased up to 15 cmH_2O. CPAP was delivered on an as-needed basis. When respiratory parameters improved, CPAP support was gradually reduced, with a progressive increase of time off CPAP, until discontinuation.

Non-invasive positive pressure ventilation

Non-invasive positive pressure ventilation (NPPV) was delivered using a portable ventilator (HAMILTON-T1 ventilator, Hamilton Medical, USA) set on the pressure support (PS) ventilation mode. PS was initially titrated to a moderate tidal volume (5-6 mL/kg of ideal body weight). The ventilator setting was then readjusted depending on the arterial blood gas (ABG) data to ensure satisfactory, but not necessarily optimal, gas exchange while protecting the lungs from the risk of ventilator-induced lung injury (VILI). The PS levels never exceeded 12 cm H_2O. Positive end-

expiratory pressure (PEEP) was usually set at 5 cmH_2O. Its levels were increased by 1-2 cm H_2O, without ever exceeding 10 cmH_2O. Supplemental oxygen was added to the ventilator circuit. The oxygen flow rate was set to achieve arterial $SaO_2 \geq 92\%$. The NIV device employed in our intermediate respiratory care unit (IRCU) was equipped with a full-face mask. In case of distress caused by wearing this mask, patients received a mild sedation (morphine sulphate 2.5-10 mg intravenously).

Criteria for intubation

Criteria for intubation were cardiac or respiratory arrest; inability to protect the airway; coma or psychomotor agitation; unmanageable secretions or uncontrolled vomiting; life threatening arrhythmias or electrocardiographic signs of ischemia; hemodynamic instability defined as systolic arterial pressure < 90 mmHg despite adequate filling or use of vasoactive agents; intolerance to all interfaces; dyspnoea during non-invasive respiratory support (NIRS), respiratory rate > 30 breaths/min; peripheral oxygen saturation (SpO_2) below 92% during NIRS, acidosis with a pH < 7.35.

Intermediate respiratory care unit organization

Nurse to patient ratios varied from a maximum of 1:4—during both days and nights—to a minimum of 1:8 during days or 1:12 during nights. In three of the seven hospitals where the study was conducted, the medical staff treating COVID-19 patients with NIRS was an ad-hoc multidisciplinary team, mainly formed by internists, pulmonologists, emergency physicians, cardiologists, anaesthesiologists/ICU physicians, while in the other four hospitals the medical team was the same as before COVID-19 pandemic. NIRS was mainly prescribed by anaesthesiologists actively working with the *ad hoc* COVID-19 ward team and, occasionally, by pulmonologists, emergency doctors or consulting anaesthesiologists, depending on the hospital. The personnel were

adequately trained for NIRS; those who were not, received a short-organized training during the pandemic. Ward monitoring included SpO_2, non-invasive blood pressure, ECG applied continuously or at a defined time point, depending on the severity of the patient. Blood gas analysis was performed when clinically relevant.
